# Gender-specific epidemiology of diabetes: a representative cross-sectional study

**DOI:** 10.1186/1475-9276-8-6

**Published:** 2009-03-11

**Authors:** Janet F Grant, Neville Hicks, Anne W Taylor, Catherine R Chittleborough, Patrick J Phillips, The North West Adelaide Health Study Team

**Affiliations:** 1 Department of Public Health, The University of Adelaide, North Terrace, Adelaide, South Australia, 5000, Australia; 2 Department of Gender, Work and Social Inquiry, The University of Adelaide, North Terrace, Adelaide, South Australia, 5000, Australia; 3 Population Research and Outcome Studies, SA Health, 11 Hindmarsh Square (PO Box 287), Adelaide, South Australia, 5000, Australia; 4 Endocrine and Diabetes Service, The Queen Elizabeth Hospital, Woodville Road, Woodville, South Australia, 5011, Australia

## Abstract

**Background:**

Diabetes and its associated complications are part of a chronic disease global epidemic that presents a public health challenge. Epidemiologists examining health differences between men and women are being challenged to recognise the biological and social constructions behind the terms 'sex' and/or 'gender', together with social epidemiology principles and the life course approach. This paper examines the epidemiology of a population with diabetes from the north-west metropolitan region of South Australia.

**Methods:**

Data were used from a sub-population with diabetes (n = 263), from 4060 adults aged 18 years and over living in the north-west suburbs of Adelaide, South Australia. Eligible respondents were asked to participate in a telephone interview, a self-report questionnaire and a biomedical examination. Diabetes (undiagnosed and diagnosed) was determined using self-reported information and a fasting blood test administered to participants. Data were analysed using SPSS (Version 10.0) and EpiInfo (Version 6.0).

**Results:**

Factors associated with diabetes for both men and women were being aged 40 years and over, and having a low gross annual household income, obesity and a family history of diabetes. In addition, being an ex-smoker and having low cholesterol levels were associated with diabetes among men. Among women, having a high waist-hip ratio, high blood pressure and reporting a previous cardiovascular event or mental health problem were associated with diabetes.

**Conclusion:**

The results found that men and women with diabetes face different challenges in the management of their condition. Public health implications include a need for quality surveillance data, including epidemiological life course, social, behavioural, genetic and environmental factors. This will enrich the evidence base for health promotion professionals and allow policy makers to draw inferences and conclusions for interventions and planning purposes.

## Background

A major challenge for public health is to halt the global epidemic of diabetes and its associated complications. For epidemiologists to successfully contribute to the reduction in the prevalence of this condition, the way in which diabetes is viewed/analysed/described needs to be changed and expanded. Traditionally, epidemiology has described people with diabetes as distinguished by sex and/or gender differences. The terms 'sex' and 'gender' have been used interchangeably or exclusively as distinct constructs – often without the recognition that sex is a biological construct and gender is a social construction that embodies particular roles and expectations [1]. Krieger [2] urges epidemiologists to be more precise about sex and/or gender difference when analysing and interpreting population health data, and looking for differences in risk or protective factors. This lack of distinction may impact upon the understanding of illness by health professionals, which potentially may influence the perception of and ability to manage their condition by people with diabetes. Gender is an important factor in social and life course epidemiology.

Equity principles highlight the need for men and women to be given an equal opportunity for good health and well-being, and physiological principles recognise sex and gender as determinants of health [3-7].

A number of studies have compared the differences in health between men and women, including the comparison of those with diabetes with other related diseases or risk factors such as coronary heart disease, physical activity, depression, dietary patterns or obesity [8-12]. A report [13] from the United States of America (US) focused on diabetes as a major health issue for women, using mortality, morbidity and survey data to highlight that women may face different challenges across their life span than men, and that these may warrant more diverse prevention, management and treatment options. Other studies have explored the risk of developing diabetes and the impact of this condition on men, as well as the effect on their lifestyles [14-18].

Diabetes is a major non-communicable and chronic condition that causes a significant degree of mortality and morbidity. It has been recognized as a continuing health challenge for the twenty-first century, both in developed and developing countries, due in part to westernization of lifestyles, as well as increasing urbanization and economic development [19].

In Australia, based on National Health Survey data from 2004–05, the rate of self-reported type 2 diabetes in all ages was 1% higher for men than for women (3% compared to 2%) [20]. In South Australia (SA), from 1991 to 2003, the age-sex standardised absolute prevalence of self-reported doctor-confirmed diabetes significantly increased for both males and females (from 3.5% in 1991 to 6.7% in 2003). Of concern is the significantly higher prevalence seen among those classified as obese (6.5% in 1991 to 12.2% in 2003), and the greatest relative percentage increases over this time among younger people aged 15 to 39 years [21].

Beckles and Thompson-Reid [13] argue that a sex/gender distinction for people with diabetes is justified, particularly for women, because of the dominance of young women developing type 2 diabetes, the impact of gestational diabetes on both the mother and baby regarding the increased risk of developing type 2 diabetes in later life, and the increasing prevalence of older women with the condition due to their greater life expectancy. Similarly, the report draws attention to the often greater burden of risk factors among women, their greater risk of developing blindness and cardiovascular disease, and the poorer survival rate and quality of life of women among those with diabetes who develop ischaemic heart disease. Further, mortality and morbidity rates for women with diabetes are higher across all causes than for women without diabetes.

It is important to acknowledge that many men with diabetes may face different challenges to women on their journey through life. It is also recognised that much data are based on self-report, and that men may be less inclined to admit problems, especially if the problem is not physically limiting [15].

This work takes a step towards answering the call from MacDonald and Crawford for a population health approach that looks at men's and women's needs rationally, as 'sub-groups of the population' [22]. It compares the distribution, determinants and frequency of diabetes in males and females over a range of self-reported and biomedically measured health conditions and risk factors, to see if there are differences in their descriptive profiles, whilst acknowledging that these factors may vary across their life course. It examines the issue from a South Australian perspective, and incorporates baseline data obtained from a subset of adult cohort participants with diabetes.

## Methods

Data used for this analysis were obtained from the North West Adelaide Health (NWAH) Study, a cohort of adults aged 18 years and over, randomly selected from the northern and western regions of Adelaide between 2000 and 2003. These areas were chosen because of their relative social disadvantage compared to many other areas of South Australia [23] and their higher levels of chronic disease and risk factors [24]. The NWAH Study provides comprehensive health information for a number of chronic diseases, including diabetes and asthma. Detailed methodology has been reported previously [25-27]. Ethics approval was received from the North Western Adelaide Health Service – Ethics of Human Research Committee (refs 45/99 & 83/2002).

All households in the north western metropolitan areas of Adelaide with a telephone connected and a number listed in the Electronic White Pages directory were eligible for selection for the study. Each randomly selected household was sent an introductory letter explaining the study and advising that they could expect a telephone call in the next few weeks. To ensure random selection within the household, the adult aged 18 years and over to have the last birthday was selected and interviewed about a number of health conditions (diabetes, asthma, bronchitis, emphysema, heart attack, stroke and angina, and mental health conditions such as anxiety, depression, a stress-related or any other mental health problem) and demographics, using the Computer Assisted Telephone Interviewing (CATI) system. Respondents were then invited to attend a clinic for a health assessment at either of two major teaching hospitals. Up to ten call-backs were made to the selected household to obtain the interview, and no replacements were made for refusal or non-response.

People who agreed to take part in the study were mailed an information pack that included a questionnaire that incorporated questions on quality of life, mental health, risk factors and health service utilisation. Demographic information included age, sex, marital status, highest educational level achieved, work status, total income of the household (before tax), and country of birth.

To define alcohol risk, respondents were asked the number of standard drinks they would usually have on a weekly and daily basis. Their alcohol risk was categorised according to the criteria used in the National Heart Foundation Risk Prevalence study [28]. The physical activity questions from the Australian National Health Surveys (conducted in 1989/90, 1995 and 2001) were used in this study to also enable comparison with national data – the now recommended Active Australia questions had not been formulated when the study was initiated. Sufficient physical activity was defined as at least 150 minutes per week of walking, moderate activity or vigorous activity [29].

Informed consent was sought from participants at the commencement of the clinic visit. A number of procedures were then conducted including a fasting blood test, performed to determine cholesterol, triglyceride, glucose and glycated haemoglobin levels. People were considered to have diabetes if they reported being told by a doctor they had diabetes, or if their fasting glucose level was greater than or equal to 7.0 mmol/L [30]. Two blood pressure measurements were taken five to ten minutes apart using a standard, calibrated blood pressure sphygmomanometer, while the participant was relaxed and seated. The average of these two recorded measures were used in the analyses. Height was measured to the nearest 0.5 centimetres using a stadiometer, and weight to the nearest 0.1 kilogram in light clothing and without shoes using standard digital scales. Body mass index (BMI) was calculated as weight (kg)/height (m)^2^. Overweight was defined as BMI ≥ 25.0 and obesity as BMI ≥ 30.0 using World Health Organization guidelines [30]. Waist circumference was measured to the nearest 0.1 centimetre using an inelastic tape maintained in a horizontal plane, with the subject standing comfortably with weight distributed evenly on both feet. The measurement was taken at the level of the narrowest part of the waist. The mean of three measurements was calculated. Hip circumference was also measured using an inelastic tape, at the level of the maximum posterior extension of the buttocks. Three measurements were taken and the average of the three was calculated. A high waist-hip ratio was defined as > 1.0 for males and > 0.85 for females [31].

### Statistical analyses

The data were weighted to the 1999 Estimated Resident Population [32] by age group, sex, region and probability of selection in the household, to ensure that the sample was representative of the population in the northern and western regions of Adelaide. The data were analysed using SPSS (Version 10.0) [33] and EpiInfo (Version 6.0) [33,34]. Weighted frequencies and general tables were produced.

Bivariate odds ratios (OR) examined the association of diabetes with demographic, risk factor and comorbidity variables separately for males and females at the conventional 0.05 p value level. Variables that were statistically significant at the 0.25 bivariate level [35] were entered into a multivariate logistic regression analysis to examine the variables independently associated with diabetes among males and females from the northern and western regions of Adelaide. Non-significant variables (p value greater than or equal to 0.05) were subsequently omitted in the modelling process until a satisfactory model was obtained. Once a satisfactory multivariate model was obtained, tests for interaction were conducted based on the likely combinations of the independent variables. Interaction terms were entered into the final multivariate model and were determined if a statistically significant improvement in the model was obtained. The presence of confounders was also assessed during the multivariate modelling process. The likelihood of confounding was assessed based on previous findings in the literature any dramatic changes in the odds ratios during the multivariate analysis [36]. If confounding existed, then the variable was removed from the model.

## Results

Of the total eligible sample (n = 8213), 71.2% (n = 5850) took part in the initial telephone interview and 49.4% (n = 4060) attended the clinic. The response rate for attendance at the clinic among those who were interviewed was 69.4%, where 1988 were males and 2071 were females. The overall prevalence of diabetes was 6.0% (95% CI 5.8 to 7.8). The prevalence of diabetes among males was 7.2% (95% CI 6.1 to 8.4) and 5.8% (95% CI 4.9 to 7.0) among females.

Table 1 describes the demographic profile of the study population, stratified by gender. Tables 2 and 3 describe the demographic, health condition and risk factor related variables tested for association with diabetes among men and women respectively.

**Table 1:** Demographic characteristics of NWAH Study participants

	**Men**		**Women**	
**Variable**	**n**	**%**	**n**	**%**

**Age group**				
18 to 29 years	512	25.7	484	23.4
30 to 39 years	387	19.5	381	19.5
40 to 49 years	378	19.0	377	18.2
50 to 59 years	284	48.6	300	14.5
60 to 69 years	207	10.4	226	10.9
70 years and over	221	11.1	302	14.6
**Area of residence**				
Western suburbs	901	45.3	952	46.0
Northern suburbs	1088	54.7	1119	54.0
**Highest education level obtained**				
Secondary	709	35.6	1043	50.4
Trade/Apprenticeship/Certificate/Diploma	966	48.6	675	32.6
Bachelor degree or higher	235	11.8	239	11.5
Not stated	79	4.0	114	5.5
**Gross annual household income**				
Up to $20,000	366	18.4	536	25.9
$20,001–40,000	526	26.5	482	23.3
$40,001–60,000	490	24.7	409	19.7
More than $60,000	499	25.1	493	23.8
Not stated	106	5.3	152	7.3
**Country of birth**				
Australia	1389	69.8	1477	71.3
UK or Ireland	312	15.7	334	16.1
Europe	179	9.0	153	7.4
Asia, Other	104	5.2	88	4.2
Not stated	5	0.3	20	1.0
**Marital status**				
Married or living with partner	1249	62.8	1277	61.6
Separated or divorced	152	7.6	178	8.6
Widowed	50	2.5	182	8.8
Never married	529	26.6	410	19.8
Not stated	8	0.4	25	1.2
**Work status**				
Full time employed	1065	53.6	472	22.8
Part time or casual employed	207	10.4	521	25.2
Unemployed	105	5.3	68	3.3
Home duties or retired	409	20.6	831	40.1
Student/Other	187	9.4	145	7.0
Not stated	15	0.8	34	1.6
Total	1988	100.0	2071	100.0

**Table 2:** Bivariate Odds Ratios for variables tested for association with diabetes among men

**Variable**	**n**	**%**	**OR**	**(95% CI OR)**	**p value**
**DEMOGRAPHIC**					
**Age group**					
18 to 39 years	12/898	1.3	1.00		
40 to 59 years	51/662	7.7	5.77	(2.95 – 11.50)	**<0.001**
60 years and over	80/428	18.8	13.99	(7.32 – 27.32)	**<0.001**
**Area of residence**					
Western suburbs	65/900	7.3	1.00		
Northern suburbs	77/1088	7.1	0.98	(0.69 – 1.40)	0.98
**Highest education level obtained**					
Secondary	48/709	6.8	1.00		
Trade/Apprentice/Certificate/Diploma	78/966	8.1	1.19	(0.81 – 1.76)	0.40
Bachelor degree or higher	8/235	3.6	0.50	(0.22 – 1.12)	0.10
**Gross annual household income**					
Up to $20,000	51/366	14.0	1.00		
$20,001–40,000	49/526	9.4	0.67	(0.43 – 1.03)	0.07
$40,001–60,000	19/490	3.9	0.28	(0.16 – 0.49)	**<0.001**
More than $60,000	11/499	2.3	0.16	(0.08 – 0.32)	**<0.001**
Not stated	11/106	10.6	0.74	(0.35 – 1.54)	0.49
**Country of birth**					
Australia	81/1389	5.8	1.00		
UK or Ireland	31/312	10.1	1.70	(1.08 – 2.68)	**0.02**
South-Eastern/Eastern Europe	25/179	13.8	2.39	(1.45 – 3.94)	**<0.001**
Asia, Other	5/104	5.0	0.82	(0.29 – 2.17)	0.85
**Marital status**					
Married or living with partner	112/1249	9.0	1.00		
Separated or divorced	16/152	10.7	1.17	(0.65 – 2.09)	0.67
Widowed	6/50	12.5	1.34	(0.50 – 3.34)	0.68
Never married	8/529	1.4	0.17	(0.08 – 0.36)	**<0.001**
**Work status**					
Full time employed	42/1065	4.0	1.00		
Part time or casual employed	13/207	6.1	1.59	(0.80 – 3.13)	0.21
Unemployed	3/105	2.7	0.72	(0.18 – 2.49)	0.79
Home duties or retired	70/409	17.0	4.34	(2.86 – 6.60)	**<0.001**
Student/Other	13/187	6.9	1.76	(0.88 – 3.47)	0.12
**SELF REPORTED RISK FACTORS**					
**Smoking status**					
Non smoker	41/839	4.9	1.00		
Ex-smoker	74/600	12.4	2.52	(1.67 – 3.82)	**<0.001**
Current smoker	27/542	5.0	1.02	(0.60 – 1.72)	0.96
**Alcohol risk**					
Non drinker, no risk	122/1597	7.6	1.00		
Low risk	11/233	4.8	0.62	(0.31 – 1.20)	0.17
Intermediate to very high risk	9/145	6.4	0.81	(0.38 – 1.69)	0.68
**Family history of diabetes**					
No	74/1398	5.3	1.00		
Yes	69/590	11.7	2.21	(1.55 – 3.15)	**<0.001**
**Family history of heart disease**					
No	76/1025	7.5	1.00		
Yes	66/962	6.9	0.93	(0.65 – 1.32)	0.72
**Family history of stroke**					
No	98/1342	7.3	1.00		
Yes	44/645	6.9	0.93	(0.64 – 1.37)	0.79
**Physical activity – *sufficient time***					
**(at least 150 minutes of walking, moderate or vigorous physical activity per week)**					
No/Insufficient physical activity	78/848	9.0	1.00		
Sufficient physical activity	66/1136	5.8	0.61	(0.43 – 0.85)	**0.004**
**MEASURED RISK FACTORS**					
**Body Mass Index**					
Acceptable	16/589	2.8	1.00		
Overweight	55/868	6.3	2.33	(1.29 – 4.28)	**0.004**
Obese	72/515	13.9	5.15	(2.88 – 9.33)	**<0.001**
Underweight	0/15	-	-	-	**-**
**Waist:hip ratio (>1.0 men, >0.85 women)**					
No	110/1834	6.0	1.00		
Yes	32/153	20.9	3.49	(2.22 – 5.45)	**<0.001**
**High blood pressure (≥ 140/90 mmHg)**					
No	64/1401	4.6	1.00		
Yes	78/586	13.3	2.91	(2.04 – 4.17)	**<0.001**
**High total cholesterol (≥ 5.5 mmol/L)**					
No	104/1274	8.2	1.00		
Yes	37/682	5.4	0.66	(0.44 – 0.99)	**0.05**
**CO-MORBIDITIES**					
**Cardiovascular disease (ever been told by a doctor)**					
No	111/1849	6.0	1.0		
Yes	32/137	23.1	3.89	(2.47 – 6.10)	**<0.001**
**Mental health disorder (ever been told by a doctor)**					
No	127/1792	7.1	1.00		
Yes	15/185	7.9	1.14	(0.63 – 2.05)	0.74
**COPD (ever been told by a doctor)**					
No	106/1338	7.9	1.00		
Yes	37/648	5.6	0.72	(0.48 – 1.08)	0.11
**Asthma (ever been told by a doctor)**					
No	124/1773	7.0	1.00		
Yes	18/213	8.5	1.21	(0.70 – 2.07)	0.56

OR Odds Ratio; CI Confidence Intervals

**Table 3:** Bivariate Odds Ratios for variables tested for association with diabetes among women

**Variable**	**n**	**%**	**OR**	**(95% CI OR)**	**p value**
**DEMOGRAPHIC**					
**Age group**					
18 to 39 years	11/861	1.3	1.00		
40 to 59 years	34/677	5.1	3.99	(1.9 – 8.3)	**0.001**
60 years and over	75/529	14.2	11.10	(5.66 – 22.31)	**<0.001**
**Area of residence**					
Western suburbs	53/952	5.6	1.00		
Northern suburbs	67/1115	6.0	1.08	(0.73 – 1.59)	0.76
**Highest education level obtained**					
Secondary	80/1043	7.6	1.00		
Trade/Apprentice/Certificate/Diploma	29/675	4.3	0.56	(0.35 – 0.88)	**0.01**
Bachelor degree or higher	5/239	2.0	0.27	(0.10 – 0.71)	**0.005**
**Gross annual household income**					
Up to $20,000	66/536	12.4	1.00		
$20,001–40,000	22/482	4.6	0.37	(0.22 – 0.62)	**<0.001**
$40,001–60,000	8/409	2.0	0.16	(0.07 – 0.35)	**<0.001**
More than $60,000	10/493	1.9	0.16	(0.08 – 0.34)	**<0.001**
Not stated	15/152	9.6	0.80	(0.43 – 1.49)	0.55
**Country of birth**					
Australia	67/1477	4.5	1.00		
UK or Ireland	36/334	10.6	2.38	(1.52 – 3.69)	**<0.001**
South-Eastern/Eastern Europe	13/153	8.5	1.87	(0.96 – 3.59)	0.07
Asia, Other	2/88	2.8	-	-	-
**Marital status**					
Married or living with partner	73/1277	5.7	1.00		
Separated or divorced	14/178	7.9	1.38	(0.73 – 2.57)	0.37
Widowed	27/182	15.1	2.60	(1.58 – 4.24)	**<0.001**
Never married	6/410	1.5	0.26	(0.10 – 0.62)	**<0.001**
**Work status**					
Full time employed	9/472	2.0	1.00		
Part time or casual employed	11/521	2.0	1.11	(0.42 – 2.93)	0.998
Home duties or retired	92/831	11.1	5.81	(2.81 – 12.43)	**<0.001**
Unemployed	3/68	4.2	-	-	-
Student/Other	3/145	2.1	-	-	-
**SELF REPORTED RISK FACTORS**					
**Smoking status**					
Non smoker	61/1070	5.7	1.00		
Ex-smoker	39/543	7.2	1.26	(0.81 – 1.94)	0.32
Current smoker	18/443	4.1	0.71	(0.40 – 1.25)	0.27
**Alcohol risk**					
Non drinker, no risk	60/552	10.9	1.00		
Low risk	53/1398	3.8	0.35	(0.23 – 0.52)	**<0.001**
Intermediate to very high risk	3/99	3.3	-	-	-
**Family history of diabetes**					
No	58/1313	4.4	1.00		
Yes	62/754	6.6	1.86	(1.27 – 2.74)	**0.001**
**Family history of heart disease**					
No	46/933	4.9	1.00		
Yes	75/1134	8.3	1.34	(0.91 – 1.99)	0.15
**Family history of stroke**					
No	63/1277	4.9	1.00		
Yes	58/790	7.3	1.49	(1.01 – 2.18)	**0.04**
**Physical activity – *sufficient time***					
**(at least 150 minutes of walking, moderate or vigorous physical activity per week)**					
No/Insufficient physical activity	71/10070	6.6	1.00		
Sufficient physical activity	51/991	5.1	0.76	(0.53 – 1.11)	0.15
**MEASURED RISK FACTORS**					
**Body Mass Index**					
Acceptable	13/803	1.7	1.00		
Overweight	31/618	5.1	3.10	(1.55 – 6.30)	**<0.001**
Obese	73/616	11.8	7.32	(3.90 – 13.99)	**<0.001**
**Waist:hip ratio (>1.0 men, >0.85 women)**					
No	45/1553	2.9	1.00		
Yes	76/513	14.8	5.11	(3.43 – 7.63)	**<0.001**
**High blood pressure (≥ 140/90 mmHg)**					
No	50/1568	3.2	1.00		
Yes	70/499	14.1	4.40	(2.97 – 6.52)	**<0.001**
**High total cholesterol (≥ 5.5 mmol/L)**					
No	73/1277	5.7	1.00		
Yes	46/759	6.1	1.06	(0.71 – 1.58)	0.84
**CO-MORBIDITIES**					
**Cardiovascular disease (ever been told by a doctor)**					
No	86/1951	4.4	1.00		
Yes	35/113	30.6	7.03	(4.44 – 11.1)	**<0.001**
**Mental health disorder (ever been told by a doctor)**					
No	82/1695	4.8	1.00		
Yes	38/357	10.7	2.20	(1.44 – 3.01)	**<0.001**
**COPD (ever been told by a doctor)**					
No	90/2465	6.1	1.00		
Yes	31/602	5.1	0.84	(0.54 – 1.30)	0.47
**Asthma (ever been told by a doctor)**					
No	98/1781	5.5	1.00		
Yes	23/286	8.0	1.46	(0.89 – 2.39)	0.15

OR Odds Ratio; CI Confidence Intervals

For men with diabetes, high statistically significant variables included age over 40 years, being on a low gross annual household income, having been born in South Eastern/Eastern Europe, undertaking home duties or being retired, being an ex-smoker, having a family history of diabetes, being obese, having a high waist-hip ratio and blood pressure, and reporting cardiovascular disease.

For women with diabetes, high statistically significant variables included age over 60 years, being on a low gross annual household income, having been born in the United Kingdom/Ireland, being widowed, undertaking home duties or being retired, being overweight or obese, having a high waist-hip ratio and blood pressure, and reporting cardiovascular disease and at least one mental health condition.

Table 4 shows the multivariate associations. There were four variables that were statistically significantly associated with diabetes for both men and women with diabetes. Both groups were more likely to be aged 40 years and over, to be obese and to have a family history of diabetes, whilst being less likely to earn more than $60,000.

**Table 4:** Multivariate Odds Ratios for variables associated with diabetes among men and women

	**MEN**			**WOMEN**		
**Variable**	**OR**	**(95% CI OR)**	**p value**	**OR**	**(95% CI OR)**	**p value**

**Age group**						
18 to 39 years	1.00			1.00		
40 to 59 years	5.28	(2.70 – 10.30)	**<0.001**	3.28	(2.01 – 5.35)	**<0.001**
60 years and over	14.58	(7.10 – 29.93)	**<0.001**	7.08	(4.17 – 12.01)	**<0.001**
**Gross annual household income**						
Up to $20,000	1.00			1.00		
$20,001–40,000	1.31	(0.81 – 2.12)	0.27	1.03	(0.72 – 1.46)	0.88
$40,001–60,000	0.73	(0.38 – 1.38)	0.33	0.65	(0.40 – 1.07)	0.09
More than $60,000	0.39	(0.19 – 0.84)	**0.02**	0.49	(0.29 – 0.85)	**0.01**
**Family history of diabetes**						
No	1.00			1.00		
Yes	3.66	(2.47 – 5.41)	**<0.001**	2.78	(2.10 – 3.70)	**<0.001**
**Body Mass Index**						
Acceptable	1.00			1.00		
Overweight	1.45	(0.80 – 2.64)	0.221	1.49	(0.95 – 2.33)	0.08
Obese	4.32	(2.39 – 7.83)	**<0.001**	3.34	(2.15 – 5.19)	**<0.001**
**Smoking status**						
Non smoker	1.00					
Ex-smoker	1.56	(1.01 – 2.42)	**0.05**			
Current smoker	1.33	(0.77 – 2.31)	0.31			
**High total blood cholesterol (>5.5 mmol/L)**						
No	1.00					
Yes	0.50	(0.33 – 0.76)	**0.001**			
**Alcohol risk**						
Non drinker, no risk				1.00		
Low risk				0.47	(0.34 – 0.65)	**<0.001**
Intermediate to very high risk				0.93	(0.49 – 1.74)	0.81
**Waist:hip ratio (>1.0 men, >0.85 women)**						
No				1.00		
Yes				2.80	(1.81 – 4.31)	**<0.001**
**High blood pressure (>140/90 mmHg)**						
No				1.00		
Yes				2.50	(1.64 – 3.83)	**<0.001**
**Cardiovascular disease**						
No				1.00		
Yes				4.03	(0.00 – 32.0)	**<0.001**
**Mental health disorder**						
No				1.00		
Yes				1.99	(1.26 – 3.14)	**0.003**

OR Odds Ratio; CI Confidence Intervals

Further, men with diabetes were statistically significantly more likely than men without diabetes to be an ex-smoker, and statistically significantly less likely to have high cholesterol.

The multivariate model for women shows that women with diabetes were statistically significantly more likely than women without diabetes to have a high waist-hip ratio, high blood pressure, and to report a past cardiovascular event (heart attack, stroke or angina) and/or a mental health problem (anxiety, depression, stress-related and/or other mental health problem), whilst significantly less likely to be a low risk drinker.

## Discussion

These local results support recent studies from the US and Canada that found gender differences in health and socioeconomic inequalities [37], suggesting the value of models that include a wide range of health and health-determinant variables whilst affirming the need to examine gender differences in health more closely.

Firstly in examining the shared variables for both men and women, it was found that older age, a family history of diabetes, low gross annual household income and obesity remained significant in the final multivariate models.

It has been well established that both age (as a social structural factor) and family history of diabetes (as a genetic/shared environment factor) are risk factors for developing the condition. Whilst neither of these factors can be altered by an individual, early knowledge may help prevent or at least delay the onset of diabetes. This could also have benefits when looking at this in the long term, if the condition could be prevented in one generation, providing a lessening of risk in their offspring.

Similarly a low household income, as another social structural factor, was consistent in both groups and it is acknowledged that people on lower incomes generally have poorer health than those on higher incomes. Therefore, improving the social conditions of a household may not only assist those currently faced with decreasing health and wellbeing, but future generations to come. This may be particularly salient for women who traditionally had less opportunity to study and therefore less access to secure, well paid employment. Women therefore are often cast in a support role, often working in part-time or casual employment due to their need to move in and out of the workforce to have and look after children, as well as provide care to ageing family members [38]. Increasingly, both sexes are being faced with the loss of permanency in work and the demands of maintaining income with balancing increasing expenditure [39]. The high divorce rate in Australia and other western societies compounds this problem, with the majority of single parents being female struggling with the additional stress of increased work and child-related commitments [40]. Men may also struggle with marriage and family breakdown, with resulting poorer mental and physical health (including impaired glucose metabolism) and economic hardship from altered circumstances [41]. Similarly, many families with both parents present are struggling with being time-poor with work and life demands, and economically challenged with rising housing, food and petrol prices, which may lead to difficulties finding the resources to sufficiently exercise and eat well.

Weight gain resulting from these factors may then lead to obesity, a lifestyle and socioeconomic factor that is another strong predictor of diabetes, hence the term "diabesity". The prevalence of obesity is generally higher amongst those with lower socioeconomic characteristics [42]. Action to combat rising obesity in many countries will assist the diabetes cause. Lieberman argues that modernisation and westernisation of lifestyles that were once physically active, and globalised availability of food that is dense in calories, high in sugar and fat and low in fibre, have led to the epidemic of obesity among children and adults in both developing and developed countries [19]. Beer [43] estimates that 80% of the prevalence of diabetes can be attributed to overweight and obesity.

In Australia, it has been estimated that 16% of men and 17% of women aged 18 years and over were obese (BMI > 30) in 2001 [44]. On a local note, of recent concern is the greatest relative percentage increase in diabetes seen in obese people less than 60 years of age, from a recent examination of annual data collected in South Australia from 1993 to 2001 [45], possibly leading to an increase in the expected prevalence. Self-reporting BMI may under-estimate the problem, given that people tend to over-report their height and under-report their weight [46]. Evidence from cohort participants in the NWAH Study (n = 4058) found that 26.0% of men and 30.0% of women were obese (clinically measured BMI at ≥ 30). It is acknowledged that ethnicity and socioeconomic factors may impact on this growing problem, and this is being examined through the NWAH Study, through life-course influences, including parents' country of birth and occupation history.

These socioeconomic and biomedical influences are seen in the remaining significant variables from the multivariate analyses for each gender in this study. For men, there were two factors: protective influence of normal total blood cholesterol (which may be due to existing cholesterol-lowering pharmacotherapy) and a previous history of smoking. The latter may be because men with diabetes may have been more likely than men without diabetes to have been advised to stop smoking by their medical practitioners, due to their increased CVD risk. The predominant factors differ for women – their significant factors are a lower alcohol risk, a high waist/hip ratio, high blood pressure, and self-reported cardiovascular and mental health co-morbidities. This suggests that 'one size fits all' policies and health promotion interventions may not adequately take into account the different lifecourses that men and women may have.

These findings are consistent with recent studies that examined gender differences in the psychosocial, structural and behavioural determinants of health [16,47]. They found that social structural factors such as age, family structure, education, income, etc significantly impact on both genders, although their effects differed for each. Looking at gender and lifestyle factors, the study found that physical activity, smoking and alcohol consumption had higher significance for men, whilst body weight was more significant for women. Equally important for both genders were psychosocial factors, such as chronic stressors, psychological resources, and childhood/life events – however, their effect was found to be generally stronger for women than for men. These disparities may be from a higher rate of self-reported ill-health, better detection or an higher rate of events.

For women, central adiposity, as measured by a high waist hip ratio, suggests that physical activity interventions may need to be tailored to this body area, as well as overall fitness. There is a tendency for both men and women to gain weight during the middle years of age, at the same time that earlier lifestyle choices start to impact on their health [48]. For women, this age group also faces the loss of protective hormonal factors through menopause. Therefore, at an individual level, there is an ongoing need to continue to emphasise the benefits of a healthy diet and to possibly increase the use of short-term pharmacotherapy to help reduce high blood glucose levels and high cholesterol, and to reduce the risk of diabetes-related complications. However, this message may need to be tailored to better fit with the expectations and lifestyle of each gender and age group. Much work is also being undertaken internationally to encourage policy and population-level changes, to provide healthier choices as the most economical and easiest option.

As previously alluded to, there is a growing prevalence of diabetes among younger people and therefore this population group should be targeted to prevent or delay ill-health, with a focus on cultural and age-appropriate interventions that target smoking, obesity, eating disorders, and lack of physical activity in communities and schools at an individual level, as well as addressing interventions and policies that assist in supporting low socioeconomic families. From a mental health and wellbeing viewpoint, there is also a need for different approaches that recognise the need for ongoing support of family, friends, counselling and education, that is balanced with the need for growing independence of this generation.

More research is needed on cardiovascular and risk-factor related factors, particularly diet and obesity, and for cultural and age appropriate interventions and recognition of the socioeconomic position of men and women with diabetes, with regard to barriers to adequate physical activity, self-care, access to quality diabetes services and health education. High blood pressure, retained in the final model for women, warrants further research regarding barriers to reaching and maintaining healthy levels. It is hoped that information collected on self-reported blood pressure status compared to measured levels, and the prescription and usage of blood pressure medication, may provide clues to this variation.

There is ongoing support for opportunistic screening of men and women in their middle years, to detect previously undiagnosed diabetes and complications, and to provide ongoing support. Support is needed to prevent or at least delay the development and related complications for all people, but particularly older people, as this will add to an already strained formal and informal care system made up of family, friends, community and state-based services.

The public health implications for both men and women with diabetes include a call for better surveillance information, as well as longitudinal data, to explore the relationships between social, behavioural, genetic and environmental factors. Policies are needed that will strengthen the co-ordination of diabetes and non-diabetes specific services at the broader community level, including the provision of welfare, access and transport.

### Limitations

There are a number of limitations that must be taken into account when reviewing the data from the NWAH Study. Using the telephone to conduct the interviews and Electronic White Pages as the sampling frame can be seen as a limitation of the study and can potentially produce biased estimates because it excludes people who do not have a telephone connected or are not listed in the White Pages [50]. However, monitoring of telephone usage in Adelaide during recruitment of cohort participants suggests that this was not expected to adversely influence results.

When compared to Census data, there was significantly less younger people (<40 years) and more older people (40+ years) who participated in the study [50]. However, other South Australian studies using different sampling methods have produced very similar results regarding diabetes prevalence [45]. This cohort does not include people residing in institutions, the majority of whom are elderly women. Neither does it include those people from a non-English speaking background who could not communicate sufficiently well with the telephone interviewer and who could not answer questions at the initial recruitment stage, although every effort was made to encourage family members to assist in translating.

The small number of Aboriginal and/or Torres Strait Islander people (n = 20) recruited in this cohort mean that no association or causality inferences can be made on their data. Furthermore, it is acknowledged that much of the data obtained are based on self-report to health-related questions, and may therefore contain an element of bias.

Finally, it should be noted that this is cross-sectional data and therefore the associations cannot be interpreted as causal.

## Conclusion

This paper compared men and women with diabetes across a comprehensive range of self-reported and biomedically measured variables relating to demographics, risk factors and co-morbidities. It found that the biological (sex) and socioeconomic (gender) characteristics of men and women with diabetes are different yet equally important, providing a challenge to a range of health professionals and associated government and non-government organisations. A number of studies are looking at each sex/gender as a research area, such as the Australian Longitudinal Study on Women's Health and the Florey Adelaide Male Ageing Study. However the NWAH Study is in a unique position to be able to compare men and women, as well as contribute knowledge and substantiate findings in both areas. This study will contribute to the growing body of knowledge about chronic disease aetiology since it developed into a longitudinal study in 2004. Through the study design, which is based on tracking participants along a continuum of disease including those at risk, those at the early stages of disease and those developing complications, changes in health status and risk behaviours will be able to be tracked and valuable information gained for use by public health professionals and researchers.

## Competing interests

The authors declare that they have no competing interests.

## Authors' contributions

JFG managed the original project from which the data were obtained and wrote the article text. NH, AWT and CRC provided ongoing advice on analysis and drafting. PJP commented on final drafts. All authors have read and approved the final manuscript.
